# Longitudinal Relationships between Social Support and Posttraumatic Growth among Adolescent Survivors of the Wenchuan Earthquake

**DOI:** 10.3389/fpsyg.2017.01275

**Published:** 2017-07-28

**Authors:** Xuji Jia, Xia Liu, Liuhua Ying, Chongde Lin

**Affiliations:** ^1^Institute of Developmental Psychology, School of Psychology, Beijing Normal University Beijing, China; ^2^Department of Psychology, Zhejiang Sci-Tech University Hangzhou, China

**Keywords:** adolescent survivors, cross-lagged model, posttraumatic growth, social support, Wenchuan earthquake

## Abstract

This study aimed to explore the longitudinal relationships between social support and posttraumatic growth (PTG) among adolescent survivors of the Wenchuan earthquake. Follow-up assessments were conducted with 452 participants at 12, 18, and 24 months after the earthquake. The results showed that the level of social support at 12 and 18 months following the earthquake predicted subsequent PTG, but not vice versa. In addition, multi-group analyses of gender showed no gender differences between social support and PTG in the cross-lagged model. Thus, psychological interventions and care for survivors should focus on improving adolescent perceptions of social support when responding to stressful experiences.

## Introduction

On May 12, 2008, a violent earthquake of 8.0 magnitude on the Richter scale struck the town of Wenchuan, China. It is estimated that more than 60,000 people lost their lives and another 400,000 thousand were injured or missing. The earthquake also resulted in a range of negative and positive psychological consequences for survivors. The negative consequences included posttraumatic stress disorder (PTSD), depression, and anxiety (Fan et al., 2011). The positive consequences, such as, “stress-related growth,” “meaning-finding” and “posttraumatic growth,” have recently been identified (Xu and Liao, 2011). Posttraumatic growth (or PTG) is a commonly used term that reflects a change in approach that extends beyond the pre-trauma level of psychological functioning (Zoellner et al., 2008). According to Calhoun and Tedeschi (2004), PTG manifests along three dimensions: changes in the perception of self, changes in interpersonal relationships and changes in philosophy of life. These positive changes have been observed in a variety of populations that have suffered traumas related to natural disasters (Xu and Liao, 2011), accidents (Zoellner et al., 2008), bereavement (Kim et al., 2011), and diseases such as, cancer (Schroevers et al., 2010).

In the developing literature on PTG, some studies have indicated that social support is significantly increased following traumatic events and that social support is correlated with PTG (Sheikh, 2004; Zhou and Wu, 2016). A meta-analysis has also shown that there is a moderate correlation between social support and PTG, with a mean effect size of *r* = 0.26 (Prati and Pietrantoni, 2009). However, more recent studies with different samples have not found a connection between the two variables (Kilmer and Gil-Rivas, 2010; Wu et al., 2016; Hill and Watkins, 2017). For example, a study by Hill and Watkins (2017) indicated that social support was not predictive of PTG among women with ovarian cancer.

Thus far, three possible modes of association between social support and PTG have been suggested. In the first mode, social support is regarded as a critical environmental resource in the development of PTG. Schaefer and Moos (1998) argued that PTG is the outcome of a posttraumatic psychological struggle. Personal system factors (e.g., personality traits) and environmental resources (e.g., social support) combine to influence cognitive appraisal and coping responses, which subsequently predict PTG. For example, McDonough et al. (2014) claimed that social support predicted increasing levels of PTG among cancer survivors. Cao et al. (2017) demonstrated that social support might influence PTG via adaptive coping among cancer patients in China. Other studies reported that social support plays a beneficial role in the development of PTG among bereaved adults (Kim et al., 2011) and college students (Swickert and Hittner, 2009). Second, PTG is considered a coping strategy that is used to find meaning in traumatic events (Park and Folkman, 1997). Consistent with this view is the finding that people who experience greater PTG are more likely to engage in helping behaviors and to perceive more social support (Steger et al., 2008). Recent investigations have provided evidence of links between positive personal meaning and social support (Sherman and Simonton, 2012). For example, a study of older adults showed that greater meaning in life was associated with increased social support (Krause, 2007). Finally, social support may in fact be independent of PTG, as some studies have documented no significant relationships between these factors (Cryder et al., 2006; Kilmer and Gil-Rivas, 2010; Wu et al., 2016).

These mixed findings are partially attributed to methodological issues. Major studies in the past have used cross-sectional research designs, which, contrary to prospective designs, prevent the examination of a temporal association between the two variables. In addition, characteristics of different populations may also partly account for the inconsistent results. For example, compared with adults, children and adolescents are particularly vulnerable to trauma. Furthermore, children and adolescents are likely to report both traumatic experiences and growth differently from adults (Meyerson et al., 2011). To the best of our knowledge, no studies have longitudinally examined the association between social support and PTG in a sample of adolescent earthquake survivors. Identifying the relationship between these two variables may contribute to the development of effective intervention plans for earthquake survivors.

This study attempts to address the above limitations by conducting a longitudinal study of adolescent survivors of the Wenchuan earthquake. It examines the relationship between social support and PTG at three time points (i.e., 12, 18, and 24 months after the Wenchuan earthquake). We propose the following competing hypotheses: (1) social support predicts PTG at subsequent assessments; (2) PTG predicts social support at subsequent assessments. Cross-lagged structural equation modeling was used to examine these relationships.

## Methods

### Participants and procedures

Participants were randomly selected from several primary and secondary schools in the counties of Wenchuan, which were the most severely affected by the Wenchuan earthquake. This study was approved by the Research Ethics Committee of Beijing Normal University and complied with the Declaration of Helsinki involving human subjects. Prior to administering the survey, school principals and teachers signed informed consent documents, and all participants agreed to participate in this study. The survey was administered by trained research assistants. Participants were informed that taking the survey was voluntary and that they were free to withdraw from it at any time.

The first data collection was conducted 12 months after the Wenchuan earthquake, at which time 452 participants were recruited (Time 1). A subsequent assessment was conducted 6 months later, in which 438 (96.90%) adolescents participated (Time 2). The third and final assessment was conducted 24 months after the earthquake, with 421 (93.14%) adolescents completing surveys (Time 3). The retention rates did not differ with respect to gender, age, grade or ethnicity.

In the final sample, 146 (34.68%) were males, and 275 (65.32%) were females. In terms of grade level, 137 (32.54%) were in Grade 7, 168 (39.91%) were in Grade 8, 35 (8.31%) were in Grade 10, and 81 (19.24%) were in Grade 11. With respect to ethnicity, 105 (24.94%) were from the Han ethnic group, 111 (26.37%) were from the Tibetan ethnic group, 186 (44.18%) were from the local Qiang people, and 19 (4.51%) were from other ethnicities. The average age was 14.87 (*SD* = 1.69).

### Measurement

#### PTG

A modified Posttraumatic Growth Inventory (PTGI) was used to assess the level of PTG. The modified PTGI was based on an original PTGI developed by Tedeschi and Calhoun (1996) and was composed of three subscales: perceived changes in the self (9 items), the sense of relationship with others (7 items), and changed philosophy of life (6 items; An et al., 2011; Jia et al., 2015). Participants rated items on a 6-point scale from 0 to 5 (0 = “no change” to 5 = “great change”). In a study of earthquake survivors, confirmatory factor analysis with the modified scale yielded a model with fitness indices of *χ*^2^/*df* = 2.35, CFI = 0.93, RMSEA = 0.07, confirming the validity of the revised scale (An et al., 2011). In this study, the level of internal consistency ranged from 0.93 to 0.95 from Time 1 to Time 3, respectively.

#### Social support

A social support scale revised by Zou (1999) was used to assess how participants evaluated the levels of social support they received. This scale assesses support from different sources: parents (4 items), teachers (4 items), and important others (4 items). For each item, the participants scored on a 5-point Likert scale (from 0 = “not at all” to 4 = “always”) the extent to which they perceived they had received support since the earthquake We used 16 items to measure support from parents, teachers and friends, with Cronbach's alpha coefficients that ranged from 0.82 to 0.89 across the three time points.

### Data analyses

We conducted a bivariate correlation analysis to examine associations between social support and PTG. As shown in Table 1, gender was correlated with three subscales of PTG from Time 1 to Time 3. Therefore, a multi-group analysis was used to test for gender differences in the relationship between social support and PTG in our cross-lagged model.

**Table 1:** Correlations among the main variables.

	**1**	**2**	**3**	**4**	**5**	**6**	**7**	**8**	**9**	**10**	**11**	**12**	**13**	**14**	**15**	**16**	**17**	**18**	**M**	***SD***
1. Gender	_																			
2. PS T1	0.09	_																	2.49	0.89
3. TS T1	−0.03	0.64^**^	_																1.97	0.82
4. FS T1	0.17^**^	0.47^**^	0.56^**^	_															2.55	0.78
5. PS T2	0.08	0.64^**^	0.38^**^	0.36^**^	_														2.39	0.86
6. TS T2	−0.01	0.42^**^	0.60^**^	0.43^**^	0.53^**^	_													1.83	0.80
7. FS T2	0.15^**^	0.32^**^	0.37^**^	0.63^**^	0.47^**^	0.58^**^	_												2.58	0.82
8. PS T3	0.09	0.48^**^	0.29^**^	0.27^**^	0.58^**^	0.37^**^	0.27^**^	_											2.48	0.84
9. TS T3	−0.04	0.31^**^	0.52^**^	0.29^**^	0.32^**^	0.60^**^	0.29^**^	0.53^**^	_										1.92	0.75
10. FS T3	0.23^**^	0.27^**^	0.31^**^	0.51^**^	0.32^**^	0.43^**^	0.61^**^	0.42^**^	0.47^**^	_									2.69	0.75
11. PTG1 T1	0.10^*^	0.32^**^	0.31^**^	0.29^**^	0.24^**^	0.24^**^	0.25^**^	0.09	0.11^*^	0.22^**^	_								3.17	1.01
12. PTG2 T1	0.12^*^	0.31^**^	0.30^**^	0.38^**^	0.24^**^	0.24^**^	0.28^**^	0.12^*^	0.13^**^	0.24^**^	0.77^**^	_							3.12	1.07
13. PTG3 T1	0.05	0.27^**^	0.27^**^	0.22^**^	0.21^**^	0.19^**^	0.18^**^	0.09	0.12^*^	0.11^*^	0.72^**^	0.68^**^	_						3.00	1.00
14. PTG1 T2	0.12^*^	0.28^**^	0.24^**^	0.27^**^	0.36^**^	0.34^**^	0.36^**^	0.19^**^	0.18^**^	0.34^**^	0.49^**^	0.44^**^	0.38^**^	_					3.02	0.98
15. PTG2 T2	0.14^**^	0.28^**^	0.26^**^	0.35^**^	0.37^**^	0.37^**^	0.46^**^	0.22^**^	0.18^**^	0.39^**^	0.43^**^	0.46^**^	0.35^**^	0.80^**^	_				2.99	1.05
16. PTG3 T2	0.07	0.25^**^	0.19^**^	0.25^**^	0.32^**^	0.31^**^	0.34^**^	0.15^**^	0.12^*^	0.26^**^	0.39^**^	0.40^**^	0.42^**^	0.74^**^	0.66^**^	_			2.84	0.99
17. PTG1 T3	0.14^**^	0.15^**^	0.22^**^	0.23^**^	0.19^**^	0.25^**^	0.22^**^	0.15^**^	0.24^**^	0.28^**^	0.46^**^	0.41^**^	0.36^**^	0.44^**^	0.41^**^	0.41^**^	_		3.07	1.00
18. PTG2 T3	0.14^**^	0.16^**^	0.20^**^	0.25^**^	0.20^**^	0.26^**^	0.27^**^	0.17^**^	0.23^**^	0.34^**^	0.45^**^	0.46^**^	0.40^**^	0.47^**^	0.46^**^	0.43^**^	0.82^**^	_	3.16	1.00
19. PTG3 T3	0.10^*^	0.12^*^	0.21^**^	0.23^**^	0.18^**^	0.25^**^	0.19^**^	0.20^**^	0.27^**^	0.27^**^	0.36^**^	0.39^**^	0.39^**^	0.41^**^	0.40^**^	0.45^**^	0.80^**^	0.80^**^	2.95	1.05

*PS, parents' support; TS, teachers' support; FS, friends' support. T1 to T2, different measurement points from Time 1 to Time 3; PTG, posttraumatic growth. PTG1 to PTG3, different subscales of posttraumatic growth representing perceived changes in the self, the sense of relationship with others and changed philosophy of life, respectively. Sex: 0, male; 1, female*.

* *p < 0.05*,

** *p < 0.01*.

To examine our hypotheses, we used structural equation modeling based on a cross-lagged model and employing Mplus 7.0 software (Muthén, and Muthén, 1998–2012). The analysis proceeded in three phases: the measurement model, the structural model and a multi-group analysis. To deal with missing data and non-normality, a maximum likelihood estimation with a mean-adjusted chi-square (MLM) was adopted. The MLM χ^2^ test statistic is also known as the Satorra–Bentler χ^2^ (Muthén, and Muthén, 1998–2012; Wang et al., 2012).

Model fit was evaluated with the comparative fit index (CFI), the Tucker–Lewis index (TLI), and the root mean square error of approximation (RMSEA). According to the recommendations of Hu and Bentler (1999), CFI values ≥ 0.90, TLI values ≥ 0.90, and RMSEA values ≤ 0.08 are all considered adequate and indicative of good fit.

The corrected and scaled χ^2^ difference, along with the change in CFI were used to test for differences in nested model fit (Satorra and Bentler, 2001). A change in CFI >0.01 indicated a poor fit (Cheung and Rensvold, 2002). A test of change in CFI is not affected by sample size, and it has a higher statistical power than the chi-square difference test. When the two results contradicted each other, we relied primarily on results of CFI differences.

## Results

### Descriptive

Table 1 presents the bivariate correlations among study variables. The results confirmed that three sources of social support and three PTG subscales were significantly associated with one another. Relationships were observed both concurrently and longitudinally, and indicate extensive relationships among these variables.

### Measurement models

Initially, we compared the fit of two measurement models. In the first measurement model, the factor loadings for the two constructs (social support and PTG) were allowed to freely estimate on the three measurement occasions (Model 1); all latent variables were correlated with one another. Error terms for each manifest variable from the different measurement times were correlated to account for the random measurement error. For example, error terms for family support at Time 1 were correlated with the same error terms at Times 2 and 3. These error terms were then correlated with each other. The first measurement model exhibited a good fit (see Table 2). The second measurement model was identical except that the factor loadings of the indicators were set as equal on the different time measurements (Model 2). If the constrained model does not significantly worsen the fit of the unconstrained model, then the constraints should be selected. This also indicates that the latent constructs are similar across the three measurement occasions (i.e., factorial invariance).

**Table 2:** Goodness-of-fit indices and model comparisons for tested models.

**Models**	**S-B χ^2^**	***df***	**RMSEA [90% CI]**	**CFI**	**TLI**
**MEASUREMENT MODELS**					
Model 1: Free loadings	154.214	102	0.035 [0.023, 0.046]	0.988	0.982
Model 2: Constrained loadings	172.363	110	0.037 [0.026, 0.047]	0.986	0.981
**STRUCTURAL MODELS**					
Model 3: Free path coefficients	210.242	114	0.045 [0.035, 0.054]	0.978	0.971
Model 4: Constraints on path coefficients	213.591	118	0.044 [0.034, 0.053]	0.979	0.972
**MULTI-GROUP ANALYSES**					
Model 5: Configural model	382.571	244	0.052 [0.042, 0.062]	0.970	0.962
Model 6: Constraints on structural coefficients across groups	394.265	256	0.051 [0.041, 0.060]	0.970	0.964

*S–Bχ^2^, scaled Satorra–Bentler χ^2^*.

The chi-square difference test revealed that Model 2 performed significantly better than Model 1 when factor loadings were set equally on three measurement occasions (the CFI difference was <0.01). Although Δ S-B χ^2^ (8) = 18.149, *p* < 0.05, as described above, when the results of the chi-square difference test and change in CFI test contradicted one another, we primarily depended on the latter. Therefore, Model 2 was preferred and retained for subsequent analyses.

### Structural models

**Figure 1:**
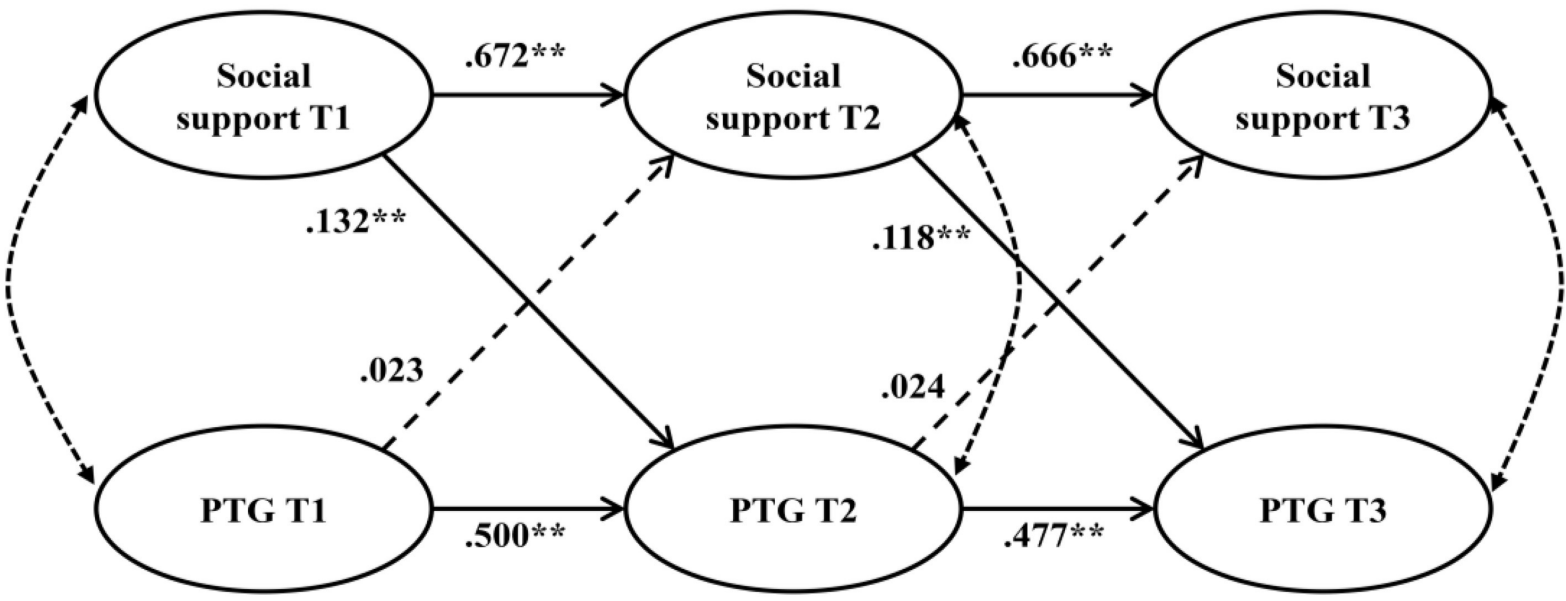
Cross-lagged model of social support and posttraumatic growth (PTG). The structural coefficients shown are standardized coefficients. Dotted lines represent covariates between constructs and/or error terms. Dashed lines represent non-significant predictions. Solid lines represent significant predictions. ^**^p < 0.01.

Next, we focused on estimating structural cross-lagged models. In cross-lagged models, one construct (e.g., social support) at Time 1 can predict the same variable at Time 2 as well as another construct (e.g., PTG) at Time 2 (cf. Figure 1). Cross-lagged effects are the effects of temporally preceding variables on another variable other after controlling for the variables stability on the different measurement occasions. Finally, we set the residual variances of social support and PTG related to each other at each measurement occasion.

A cross-lagged model (Model 3) with estimating all path coefficients freely was examined, and showed a good fit to the data (see Table 2). In the next cross-lagged model (Model 4), we set all path coefficients equally on the three measurement occasions. This model also indicated a good fit to the data (see Table 2). The difference in fit between Models 3 and 4 was non-significant, as the CFI difference was <0.01, Δ S-B χ^2^ (8) = 18.149, *p* < 0.05. Consequently, Model 4 was regarded as the final model.

In Figure 1, we see the standardized path coefficients for Model 4. The stability coefficients were 0.672 and 0.666 for social support (all *p*s < 0.01). The stability coefficients of PTG were 0.500 and 0.477 (all *p*s < 0.01). The cross-lagged effects from social support to PTG (standardized path coefficients β = 0.132 and 0.118) were significant (all *p*s < 0.01), whereas the cross-lagged effects from PTG to social support (standardized path coefficients β = 0.023 and 0.024) were non-significant.

### Multi-group analyses

Multi-group analyses were conducted to assess whether Model 4 should be run separately for each gender. Two nested models were examined, starting with the least restricted model first. Unlike the measurement model, the least restricted model (Model 5) allowed all parameters to be estimating freely across groups. This model provided a good fit (see Table 2). In Model 6, equality constraints were imposed on the stability paths, the cross-lagged paths, as well as the measurement models across both males and females. This restricted model also provided a good fit (see Table 2). A comparison of the two nested models yielded a non-significant chi-square difference, suggesting there were no differences in the model based on gender. Hence, for both males and females, the estimates of path coefficients were similar to estimates for the total sample.

## Discussion

Although many studies have indicated that social support is connected to PTG, in the case of different types of trauma, the literature has also presented mixed findings about the association. The present study examined a temporal aspect of the relationship between social support and PTG in a sample of earthquake survivors. Our main finding is that PTG is an outcome of social support and not vice versa. Social support predicted subsequent PTG from 12–24 months after the Wenchuan earthquake. This result agrees with the Schaefer and Moos (1998) model and is consistent with empirical studies on the relationship between sources of social support and PTG (Schroevers et al., 2010; McDonough et al., 2014). For example, social support from family members and friends was associated with higher PTG scores (Kimhi et al., 2009). As a result, the present study suggests that strong social support is crucial for facilitating and maintaining PTG. In addition, cognitive processing theory offers an important perspective for explaining the significance of social support (Tedeschi and Calhoun, 2004). In responding to the negative emotions following traumatic events, earthquake survivors need to feel more connected with others. Parents, teachers, and friends provide survivors with various forms of intangible support (e.g., emotional support, informational support). In addition, numerous tangible forms of assistance are also offered to cope with such stressors (Swickert and Hittner, 2009). Consequently, earthquake survivors themselves may treat others more positively over time and experience greater self-efficacy in the face of stressful events, which in turn are important to higher levels of PTG (Tedeschi and Calhoun, 2004; Cryder et al., 2006; Zhou and Wu, 2016).

Contrary to previous studies (Krause, 2007; Sherman and Simonton, 2012), we were unable to show that PTG promoted social support. In addition, contrary to models of PTG as a coping strategy, initial PTG levels reported 12 and 18 months after the Wenchuan earthquake did not predict subsequent social support levels. However, this result does not indicate that such models are false. Many theorists have framed PTG as both a coping outcome and a coping strategy (Zoellner and Maercker, 2006; Cao et al., 2017). One explanation for our inability to detect an effect of PTG on social support lies in the 24-month time frame itself. It was simply too short, as such a relationship would not likely emerge until much later (perhaps even 10 or more years after the trauma). Typically, studies describe two phases of coping with trauma, especially wide-ranging trauma (Kaniasty and Norris, 2008). In the initial phase, social support may be beneficial, helping survivors to recover from traumatic experience or grow, an outcome that has been demonstrated in many disaster studies (Sheikh, 2004; McDonough et al., 2014; Zhou and Wu, 2016). In the next stage, in light of changing and socially shared schemas, a person with a high level of PTG may come to see society more positively, and may become more likely to thrive in social relationships and generate mutual support. This outcome is consistent with social selection theory (Kaniasty and Norris, 2008). In fact, a study of PTSD showed that more severe PTSD symptoms lead to less social support at 18 to 24 months (Kaniasty and Norris, 2008). However, with respect to PTG, the specific time point where it predicts later social support, needs to be examined further in future research.

In addition, our results indicated that gender was significantly related to PTG and that females reported more PTG than did males. The magnitude of this association was similar to previous findings (Park et al., 1996; Linley et al., 2008). Why is gender associated with PTG? We believe the association of gender with PTG is related in part to the mediating influence of social support, as females are more willing to seek help from others in times of stress (Swickert and Hittner, 2009). Our data showed that females reported greater levels of support from friends than did males (*r* = 0.15–0.23) and that they did so at different time points. Thus, when coping with stressors, females may form richer social connections, which can lead to greater levels of PTG. However, when examining the cross-lagged model between social support and PTG, we found that both males and females followed the same pathway from social support to PTG. It seems that the facilitative role of social support in PTG is equal for males and females. In light of the different roles played by both global and relationship-specific support, future research should explore the relationship between the latter and PTG, as well as any associated gender differences. Ascertaining the pathways between relationship-specific social support and PTG as well as the influence or lack of influence of gender on them may yield important information relevant to promoting PTG among earthquake survivors.

Several limitations of the current study warrant mentioning. First, like trauma studies, social support and PTG were typically assessed using self-reports. It is possible that the observed relationships stemmed from a shared variance related to method. Future research should employ multiple measures from different informants, as well as additional objective measures of PTG and social support (e.g., partner ratings of relationship quality). Second, our results were constrained by its focus on adolescent survivors assessed from 12–24 months after an earthquake. Future longitudinal studies should examine the association between PTG and social support within different trauma populations and at more time points. Finally, the lack of a pre-trauma assessment of social support limited our ability to determine causality.

Despite these limitations, our results offer insight into the relationship between social support and PTG among adolescent survivors of the Wenchuan earthquake, 12–24 months after the earthquake. Furthermore, the findings in this study have important clinical implications. For instance, increased social support predicts the PTG levels for both men and women. Hence, it is necessary for clinicians and teachers to help survivors recognize those upon whom they can rely in moments of crisis, improving the perceptions of social support in the context of responding to stressful experiences.

## Ethics statement

This study was carried out in accordance with the recommendations of the Research Ethics Committee of Beijing Normal University with written informed consent from all subjects. All subjects gave written informed consent in accordance with the Declaration of Helsinki. The protocol was approved by the Research Ethics Committee of Beijing Normal University.

## Author contributions

Conceived and designed the experiments: XJ and CL. Performed the experiments: LY and XL. Analyzed the data: XJ and LY. Wrote the paper: XJ, XL, and CL.

### Conflict of interest statement

The authors declare that the research was conducted in the absence of any commercial or financial relationships that could be construed as a potential conflict of interest.
